# MiRNA expression patterns predict survival in glioblastoma

**DOI:** 10.1186/1748-717X-6-153

**Published:** 2011-11-10

**Authors:** Maximilian Niyazi, Franz Zehentmayr, Olivier M Niemöller, Sabina Eigenbrod, Hans Kretzschmar, Klaus-Schulze Osthoff, Jörg-Christian Tonn, Mike Atkinson, Simone Mörtl, Claus Belka

**Affiliations:** 1 Department of Radiation Oncology, Ludwig-Maximilians-University Munich, Marchioninistr. 15, 81377 Munich, Germany; 2 Department of Neuropathology, Ludwig-Maximilians-University Munich, Marchioninistr. 15, 81377 Munich, Germany; 3 Interfakultäres Institut für Biochemie, University of Tübingen, Hoppe-Seyler-Str. 4, 72076 Tübingen, Germany; 4 Department of Neurosurgery, Ludwig-Maximilians-University Munich, Marchioninistr. 15, 81377 Munich, Germany; 5 Helmholtz Zentrum München, Institute of Radiation Biology, Ingolstädter Landstr. 1, 85764 Neuherberg, Germany

**Keywords:** radiotherapy, glioblastoma, microRNA, methylation, prognosis

## Abstract

**Background:**

In order to define new prognostic subgroups in patients with glioblastoma a miRNA screen (> 1000 miRNAs) from paraffin tissues followed by a bio-mathematical analysis was performed.

**Methods:**

35 glioblastoma patients treated between 7/2005 - 8/2008 at a single institution with surgery and postoperative radio(chemo)therapy were included in this retrospective analysis. For microarray analysis the febit biochip "Geniom^® ^Biochip MPEA homo-sapiens" was used. Total RNA was isolated from FFPE tissue sections and 1100 different miRNAs were analyzed.

**Results:**

It was possible to define a distinct miRNA expression pattern allowing for a separation of distinct prognostic subgroups. The defined miRNA pattern was significantly associated with early death versus long-term survival (split at 450 days) (p = 0.01). The pattern and the prognostic power were both independent of the MGMT status.

**Conclusions:**

At present, this is the first dataset defining a prognostic role of miRNA expression patterns in patients with glioblastoma. Having defined such a pattern, a prospective validation of this observation is required.

## Introduction

Glioblastoma multiforme (GBM) is the most common and aggressive primary brain tumor [1]. Malignant gliomas account for approximately 70% of new cases of malignant primary brain tumors diagnosed in adults. Median age at diagnosis of primary gliomas is 64 years and malignant gliomas are more common in men than in women [2].

Currently the treatment of GBM is based on a multidisciplinary approach including surgery and adjuvant radiochemotherapy followed by maintenance chemotherapy. Concomitant and adjuvant administration of temozolomide improved 2-year survival of patients with newly diagnosed malignant glioma (mainly GBM) from 11% to 27%, 3-year survival from 4% to 16% and 5-year survival from 2% to 10% [3,4].

Despite all developments for primary and recurrent glioblastoma [3,5], there is still extreme room for further improvement since glioblastoma has a dismal prognosis for most of the patients with a high rate of local recurrences [6]. At present, several strategies may lead to an optimization: Firstly, better imaging tools as well as improved image-guidance are available [7-10] and dose escalation while sparing normal tissue has been achieved by new technical approaches such as intensity-modulated radiotherapy [11], volumetric single arc technique [12] or older techniques such as fractionated stereotactic boost/radiosurgery [13]. Secondly, in addition to an improved application of radiotherapy, the combination of radiation with targeted drugs may turn out to increase the therapeutic ratio. In this regard different targeted molecules are currently undergoing pre-clinical and clinical testing [14-20].

Closely associated with the research fields mentioned above, it is of crucial importance to gain insight into the underlying biological reasons for different patient outcomes. It is well known that the prognosis of patients with glioblastoma differs considerably on an individual scale [21].

At present only very few prognostic factors such as a higher age or a bad ECOG score have been defined. Recently, certain molecular pathways and associated biomarkers have been established as prognostic and predictive markers. In this regard, Hegi et al. identified the O6-methylguanine DNA-methyltransferase (MGMT) promoter methylation status to be a potent prognostic factor for GBM patients with a potential predictive value for the efficacy of temozolomide-based radiochemotherapy [22,23]. Additionally, IDH1 and IDH2 status have recently been introduced in GBM but their predictive role has not yet been defined though their prognostic power is well-known [24,25]. However, neither MGMT methylation nor other markers are precise enough to enable individual assessments.

The use of microRNAs (miRNAs) as tumor biomarkers has gained growing interest in the last few years. Accumulating evidence indicates that miRNA expression can be used as a prognostic and/or diagnostic marker for human cancers [26]. The miRNAs consist of 18-25 nucleotides and are a class of endogenous ribo-regulators that modulate gene expression via the RNA interference (RNAi) pathway [27]. The discovery of miRNAs dates back to 1993 when Lee et al. described a small RNA, lineage-deficient-4 (lin-4), with antisense complementarity to lin-14 involved in the regulation of developmental timing in *Caenorhabditis elegans *[28]. MicroRNA deregulation is implicated in processes such as cell proliferation, cell cycle regulation, apoptosis, invasion, glioma stem cell behavior and angiogenesis [29,30].

More than 1000 microRNAs (miRNAs) are present in the human genome. Expression is largely tissue and cell type specific, with some miRNAs considered to be housekeeping molecules. Each miRNA is predicted to target and possibly regulate multiple mRNA species. Diverse high-throughput screenings of various systems have as yet identified only a limited number of functional miRNAs [31].

Their role in regulating a great variety of targets and, as a consequence, multiple pathways, makes their use in diagnostics potentially a powerful tool to be exploited for risk assessment and prognosis and for the design of innovative therapeutic strategies [32].

With all the inherent limitations of a retrospective analysis in mind, we have determined the prognostic value of miRNA expression patterns for overall survival in 35 primary glioblastoma patients who were treated at our institution with radio(chemo)therapy following surgery. Follow-up data and MGMT methylation status were used to test whether miRNA profiles may be MGMT-independent prognostic factors for overall survival.

## Patients and Methods

All glioblastoma patients treated with surgery and postoperative radio(chemo)therapy treated between 7/2005 and 8/2008 at our institution were identified using the departmental database. Radiochemotherapy with temozolomide was applied according to the EORTC/NCIC regimen [3,4].

Our study was approved by the local ethics committee (No. 442-09), histopathologic diagnosis was confirmed by central pathology review in all cases.

Determination of MGMT promoter methylation was performed using both methylation-specific PCR and sequencing analysis as being published before [33,34].

For miRNA analysis the biochip "Geniom^® ^Biochip MPEA homo sapiens" from febit was used (Febit holding GmbH, Im Neuenheimer Feld, Heidelberg, Germany). The probes are designed as the reverse complements of all major mature miRNAs and are based on sequences as published in the current Sanger miRBase release (version 15.0 April 2010, see http://microrna.sanger.ac.uk/sequences/index.shtml) for Homo sapiens. Additional nucleotides are bound on the 5'end of each capture oligonucleotide that serve as template for the enzymatic extension in the labelling procedure. The probes are synthesized with intra-array replicates to increase the statistical confidence and to compensate for potential positional effects. The intensities of blank probes consisting only of one single "T" nucleotide are used for background corrections.

### Sample washing and detection

Total RNA was isolated from FFPE tissue sections using the QIAGEN RNeasy FFPE Kit. For each array the RNA was suspended in febit's proprietary miRNA Hybridization Buffer (25 μl per array). Hybridization was done automatically for 16 h at 42°C using the Geniom RT^®^-Analyzer. After washing the chips were labelled using the microfluidic-based primer extension assay. This assay utilizes the bound miRNAs as a primer for an enzymatic elongation with biotinylated nucleotides. The elongation was done using the Klenow polymerase I Fragment at 37°C for 15 minutes. Biotin incorporation was detected with streptavidin-phycoerythrin, in combination with febit's consecutive Signal Enhancement procedure. The feature recognition (using Cy3 lter set) and signal calculation were done automatically.

### Clustering analysis

In order to define miRNA patterns similar clusters were identified by hierarchical clustering. In this regard, a similarity matrix was generated that contained all pairwise similarities between probes or samples. The Euclidean distance was applied for the similarity measurement.

A hierarchy of clusters was then built for the similarity matrix. This hierarchy is represented as a tree, a so-called dendrogram. To compute the similarity between two clusters complete linkage (distances of all pairs of elements in both clusters) was applied.

As a final result, a heatmap was generated, i.e., a colored representation of samples and probes, ordered by their similarity with a dendrogram on top (clustering of samples) and on the right side (clustering of probes).

To detect possible clusters in rows (transcripts) and columns (samples) of the normalized expression matrix we carried out a bottom-up complete linkage clustering using the Euclidean distance a measure.

### Statistics

We performed all analyses using the Statistical Package for Social Sciences (SPSS, Ver. 19.0, SPSS Inc, Chicago, IL). Survival analyses were based on Kaplan-Meier estimates, univariate testing was performed by means of the log-rank test and Cox regression analysis was used to determine hazard ratios as well as to perform a multivariate analysis. The correlation between two dichotomous variables was assessed using Fisher's exact test. A two-tailed p-value ≤ 0.05 was considered significant.

## Results

### Patient characteristics

We examined paraffin tissue samples of a non-selected cohort of consecutively treated patients at the Ludwig-Maximilians university hospital Munich, Großhadern from 7/2005 to 8/2008. Only patients with surgery and postoperative radio(chemo)therapy were eligible. Median overall survival of the patient cohort was 530 days, median follow-up was also 530 days including early deaths (range, 16 - 1545 days). MGMT status was available in 30 cases, missing in five cases (see Table 1 for all baseline characteristics). The attribution of RPA-classes in retrospect is difficult and therefore comprises some degree of uncertainty. In 8 cases no definite assignment could be performed; the majority of patients (85.2%) had RPA class IV and V [35].

**Table 1:** Baseline patient characteristics, N = 35, TMZ - temozolomide.

Characteristic	Patients
MGMT methylation status	
• methylated	17
• not methylated	13
• unknown	5
Median follow-up (range)	530 days (16 - 1545)
Sex	
• male	22
• female	13
Median age (range)	62 (33 - 77)
Age	
• < 60 y	14
• ≥ 60 y	21
Type of resection	
• complete	18
• incomplete	17
RPA class	
• III	4
• IV	11
• V	12
• not exactly known	8
Re-RT	
• yes	4
• no	31
Concomitant TMZ	
• yes	26
• no	7
• unknown	2
Adjuvant TMZ	
• yes	19
• no	14
• unknown	2
Survival	
• long-term	20
• short-term	15

### Survival data and univariate analysis

In a first step, we determined whether a survival cutoff-value, defined by the median survival within the EORTC-NCIC trial 26981 of nearly 450 days, would clearly separate long- and short-term survivors in our analysis. The difference between long- and short-term survival was significant (p < 0.001). Median overall survival was 990 days for long-term and 267 days for short-term survivors; mean values were 1075 days and 244 days, respectively.

**Figure 1:**
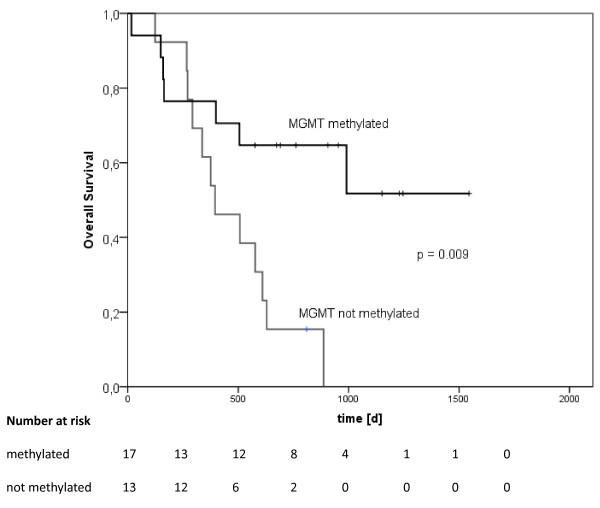
**Kaplan-Meier overall survival curves for MGMT methylated (N = 17) or MGMT not methylated (N = 13) patients**. Significant difference by log-rank test (p = 0.009).

MGMT had a significant influence in the univariate analysis on overall survival: p = 0.009, hazard ratio 3.6 (95%-CI 1.3 - 9.7) (Figure 1, Table 2). Median survival of MGMT methylated patients was not defined, MGMT not methylated patients had a median overall survival of 395 days. Mean values were 1010 days vs. 474 days. Data of 5 patients could not be evaluated due to unknown MGMT status.

**Table 2:** Univariate analysis on potential prognostic factors for overall survival after primary diagnosis, ns - not significant, CI - confidence interval, meth - methylated.

Factor	p-value (unadjusted, log-rank)	Hazard ratio	95%-CI
MGMT methylation status (not meth/meth)	0.009	3.6	1.3 - 9.7
Sex (male/female)	ns (0.4)	1.2	0.8 - 1.8
Re-RT (no/yes)	ns (0.5)	1.7	0.4 - 7.0
Concomitant TMZ (no/yes)	0.04	2.6	1.0 - 6.8
Adjuvant TMZ (no/yes)	0.001	4.1	1.7 - 9.8
Age category (< 60 y, ≥ 60 y)	ns (0.1)	0.5	0.2 - 1.2
Type of resection (complete/incomplete)	ns (0.83)	0.9	0.4 - 2.0
RPA class (V/IV/III)	ns (0.08)	IV (0.5), III (0.2)	IV (0.2 - 1.2), III (0.02 - 1.4)

The results of the univariate analysis for additional prognostic factors such as sex, age (≥ 60 y, < 60 y), concomitant/adjuvant temozolomide, RPA class, type of resection and re-irradiation are shown in Table 2. In this regard, sex, re-irradiation, type of resection and age category were non-significant factors, whereas the use of temozolomide (concomitant and adjuvant) were significant and RPA class marginally significant (p = 0.08).

### Detection of differentially expressed miRNAs

For the detection of differentially regulated miRNAs, a wide variety of measures had been proposed. These methods include quotation of mean, median, or variance, parametric t-test, non-parametric Wilcoxon-Mann-Whitney test, Empirical Bayes Statistics and the area under the receiver operator characteristics curve. For all significance tests (t-test, Wilcoxon-Mann- Whitney test and Empirical Bayes Statistics) the raw p-values are provided. However, since multiple miRNAs are tested, these p-values must be adjusted for multiple testing to control the False Discovery Rate. Significantly deregulated miRNAs were miR-3163 (fold change 2.0, p = 0.05), miR-539 (fold change 0.5, p = 0.001), miR-1305 (fold change 0.5, p = 0.05), miR-1260 (fold change 0.5, p = 0.03) and let-7a (fold change 0.3, p = 0.02, in all cases unadjusted p-values; 30 most deregulated probes).

### Correlations and multivariate analysis

**Figure 2:**
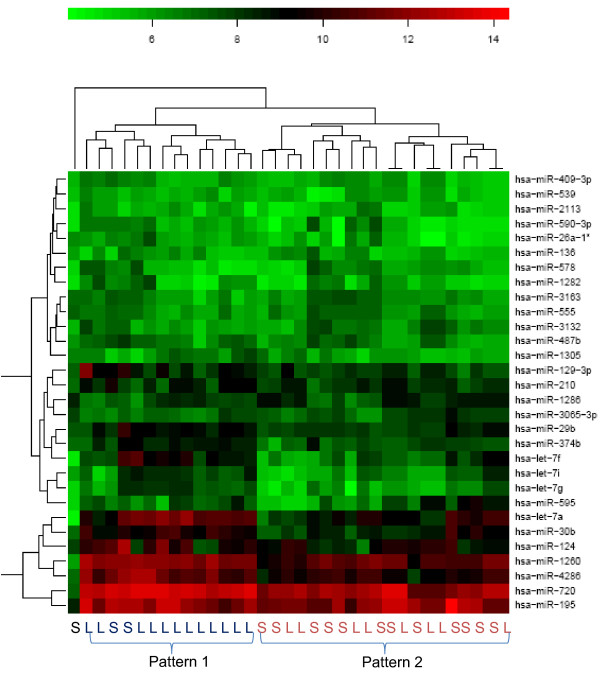
**Heatmap with dendrograms for comparison short-term (S) vs. long-term survivor (L)**.

As seen in Figure 2, there are two branches of the dendrogram, grouping 34 of the 35 samples into two categories. These two groups show different expression patterns of the 30 analyzed miRNAs (those which are maximally deregulated). Most interestingly, these two patterns correlate with survival (short-term vs. long-term survival): p = 0.01 (Fisher's exact test) but do not correlate with the MGMT status (p = 0.5).

**Figure 3:**
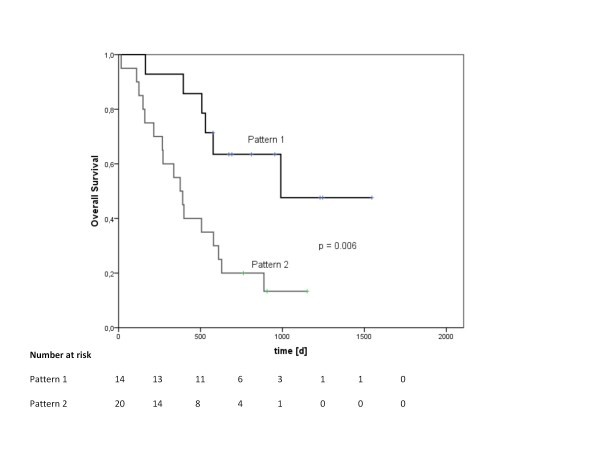
**Kaplan-Meier overall survival curves for patients with miRNA pattern 1 (N = 14) or pattern 2 (N = 20)**. Significant difference by log-rank test (p = 0.006).

In order to examine the real influence of both patterns on survival, Kaplan-Meier curves for both patterns were plotted (see Figure 3). It could be shown that pattern 1/2 was associated with increased/decreased survival, p = 0.006 in univariate analysis, hazard ratio 0.3 (95%-CI 0.1 - 0.7); median survival for pattern 1 was 990 days, for pattern 2 it was 376 days. Mean values were 1053 days (pattern 1) and 470 days (pattern 2).

Analyzing the slightly more homogeneous group of patients who received concomitant temozolomide (N = 26) still revealed a significant result in univariate analysis for the pattern 1/2 (p = 0.005, log-rank test) and there was still no correlation between MGMT methylation status of these patients and pattern 1/2 (p = 0.2).

When performing a multivariate analysis for all patients including the factors MGMT status, miRNA pattern, adjuvant temozolomide, age category and RPA class, it turns out that only adjuvant temozolomide remains a prognostic factor (p = 0.01), MGMT status and miRNA pattern lose their prognostic significance (p = 0.17 and p = 0.22).

## Discussion

In order to define new marker constellations for a more precise separation of different prognostic groups in patients with GBM we performed a miRNA array analysis including merely all currently known miRNAs.

In our analysis, two complementarily defined miRNA pattern predicted early death versus long-term survival (split at 450 days) in a significant way (p = 0.01) and this prediction was independent of the MGMT status. Thus, importantly the miRNA pattern was correlated with outcome independently of the MGMT status within the univariate analysis. This could not be proven within the multivariate analysis due to statistical limitations as the case number and the number of events was too small.

Our findings clearly indicate that complex alterations of the regulatory network involved in tumor gene expression are at least as important as a single disturbance of a single DNA repair enzyme.

At present, the role of individual miRNAs in GBM is poorly understood. MiRNAs are small noncoding regulatory RNAs that reduce stability and/or translation of fully or partially sequence-complementary target mRNAs [36]. In this sense, they are important post-transcriptional gene regulators and play an important role in response to cellular stress [37] as well as pathogenesis of cancer development and progression [38] with miR-17 and miR-184 being promising candidates for tumor progression [39].

It has been shown recently that GBMs display a distinct miRNA expression signature and a number of recent studies have linked these miRNA alterations to key hallmarks of GBM including proliferation, survival, invasion, angiogenesis and stem cell-like behavior [40,41]. Moreover, resistance to temozolomide might be associated with miRNA deregulation [42]. In this regard, Ciafre et al. studied the global expression of 245 microRNAs in GBM using a microarray technique [43] in comparison to normal brain tissue. This approach enabled the identification of miRNAs whose expression is significantly altered in tumors compared with peripheral brain areas from the same patient, including miR-221, strongly up-regulated in GBM, and a set of brain-enriched miRNAs, miR-128, miR-181a, miR-181b, and miR-181c, which were down-regulated in glioblastoma [36].

In contrast to the available data on miRNA in GBM, our approach differed significantly. Based on the hypothesis that an equally complex network of different miRNAs might regulate complex metabolic interactions regulating processes such as the therapy response in tumor cells we speculated that a fingerprinting approach using biological extremes would reveal informative prognostic data. Indeed our approach revealed a miRNA pattern being significantly associated with the outcome of the pre-defined biological extremes.

However, at present one has to bear in mind that the approach chosen is purely retrospective and does not contain a control group of uninvolved brain parenchyma. It is only useful to generate a hypothesis but not to prove a predictive character of the observed miRNA pattern. Thus it is mandatory to validate the miRNA pattern in a prospective trial.

Besides the validation of the given pattern, it is of major interest to understand to role of individual miRNAs for the biological response of GBM. Among the panel of miRNAs, there are some with relatively unknown cell cycle function, such as miR-3163, miR-1305, miR-1260. Others have already been examined on a functional level such as miR-539 which is among the factors sensing biotin and regulating holocarboxylase synthetase. This enzyme plays an essential role in catalyzing the biotinylation of carboxylases and histones. Biotinylated carboxylases are needed for the metabolism of glucose, lipids and leucine; biotinylation of histones plays important roles in gene regulation and genome stability [44]. The most prominently deregulated miRNA is let-7a: Lethal-7a was recently found to be associated with several cancers, such as lung and colon cancers. It was also proposed that let-7a may be a tumor suppressor in laryngeal cancer by inhibiting cell growth, inducing cell apoptosis and down-regulating oncogene expression; in Hep-2 cells, let-7a induced apoptosis and downregulated RAS and c-MYC protein expression without affecting the mRNA levels [45]. But none of these miRNAs has been linked to glioblastoma up to now.

## Conclusions

This study is - to our best knowledge - the first clinical miRNA screening trial in GBM patients resulting in the definition of a distinct miRNA pattern. This pattern may serve as potential new prognostic and/or predictive marker set allowing for patient stratification independent of the MGMT status. Although the trial was based on a small sample size and is limited by the retrospective character of the study, the data urgently mandate a prospective validation and additional research in order to define to biological role of miRNA alteration for the pathogenesis of glioblastoma.

## Abbreviations

CI: confidence interval, GBM: glioblastoma multiforme, MGMT: O-6-methylguanine-DNA methyltransferase, miRNA: micro ribonucleic acid, PCR: polymerase chain reaction.

## Competing interests

The authors declare that they have no competing interests.

## Authors' contributions

MN performed the critical analysis, associated statistics and wrote the manuscript. FZ collected patient data and wrote the study proposal. SE and HK provided the paraffin embedded samples. ON, KSO, JCT, SM and MA critically revised the manuscript. CB provided the idea, conception, strategy and took part in the critical analysis as well as the preparation of the manuscript. All authors read and approved the final manuscript.
